# Randomized Controlled Trial Comparing a Multidisciplinary Intervention by a Geriatrician and a Cardiologist to Usual Care after a Heart Failure Hospitalization in Older Patients: The SENECOR Study

**DOI:** 10.3390/jcm11071932

**Published:** 2022-03-30

**Authors:** Marta Herrero-Torrus, Neus Badosa, Cristina Roqueta, Sonia Ruiz-Bustillo, Eduard Solé-González, Laia C. Belarte-Tornero, Sandra Valdivielso-Moré, Olga Vázquez, Núria Farré

**Affiliations:** 1 Geriatrics Department, Hospital del Mar, 08003 Barcelona, Spain; mherrero@psmar.cat (M.H.-T.); croqueta@psmar.cat (C.R.); ovazquez@psmar.cat (O.V.); 2 Heart Failure Unit, Cardiology Department, Hospital del Mar, 08003 Barcelona, Spain; nbadosa@psmar.cat (N.B.); sruiz@psmar.cat (S.R.-B.); edsole@clinic.cat (E.S.-G.); lbelarte@psmar.cat (L.C.B.-T.); svaldivielso@psmar.cat (S.V.-M.); 3 Biomedical Research Group on Heart Disease, Hospital del Mar Medical Research Group (IMIM), 08003 Barcelona, Spain; 4 Department of Medicine, Universitat Autónoma de Barcelona, 08193 Barcelona, Spain; 5 Department of Experimental and Health Sciences, Universitat Pompeu Fabra, 08002 Barcelona, Spain

**Keywords:** geriatrician, prognosis, heart failure, all-cause hospitalization, cardiologist, frailty

## Abstract

Background: The prognosis of older patients after a heart failure (HF) hospitalization is poor. Methods: In this randomized trial, we consecutively assigned 150 patients 75 years old or older with a recent heart failure hospitalization to follow-up by a cardiologist (control) or follow-up by a cardiologist and a geriatrician (intervention). The primary outcome was all-cause hospitalization at a one-year follow-up. Results: All-cause hospitalization occurred in 47 of 75 patients (62.7%) in the intervention group and in 58 of 75 patients (77.3%) in the control group (hazard ratio, 0.67; 95% confidence interval, 0.46 to 0.99; *p* = 0.046). The number of patients with at least one HF hospitalization was similar in both groups (34.7% in the intervention group vs. 40% in the control group, *p* = 0.5). There were a total of 236 hospitalizations during the study period. The main reasons for hospitalization were heart failure (38.1%) and infection (14.8%). Mortality was 24.7%. Heart failure was the leading cause of mortality (54.1% of all deaths), without differences between groups. Conclusions: A follow-up by a cardiologist and geriatrician in older patients after an HF hospitalization was superior to a cardiologist’s follow-up in reducing all-cause hospitalization in older patients. (Funded by Beca Primitivo de la Vega, Fundación MAPFRE. ClinicalTrials.gov number, NCT03555318).

## 1. Introduction

Heart failure (HF) is a syndrome that predominantly affects older patients. At a population level, a study carried out in Spain showed that 68% of the patients with HF are over 75 years old, and 30% are over 84 years old [1]. Furthermore, HF is the leading cause of hospital admission in patients over 65 years of age [2]. After a hospitalization due to HF, the prognosis is poor, with up to 58% all-cause rehospitalization and 23.7% mortality at one-year follow-up [1,3,4,5]. The prevalence and cost associated with HF are expected to increase due to the aging population and improvements in treatment [6]. Several interventions to reduce hospitalizations in heart failure have been assessed. However, most of the studies were carried out in patients with HF with reduced ejection fraction (HFrEF) and with a mean age less than 80 years old [7].

HF in older patients has a series of characteristics that make it different from younger patients due to a confluence of several aspects. On the one hand, age per se is associated with a decline in several domains of cardiovascular function (diastolic function, LA volumes) and impaired reversed remodeling [8,9]. On the other hand, there are few treatments for HF with preserved ejection fraction (HFpEF) [10]. In patients with HFrEF, the effect of treatment in specific subgroups (frail, cognitively impaired, nursing homes residents) has not been adequately studied [11,12]. Furthermore, comorbidity, widespread in HF, increases with age and is associated with a worse prognosis [1]. However, the most remarkable difference between older and young patients is the possibility of functional impairment (sometimes reversible) and the lower capacity to react to stressors (frailty) [13]. Frailty, which is present in up to 44% of patients with HF [14,15], is independently associated with higher mortality [14,15,16,17,18] and poorer quality of life [19]. A comprehensive geriatric assessment is a multidimensional and interdisciplinary diagnostic process used to evaluate frail older adults’ medical, psychological, functional, and social aspects to develop a coordinated, individualized, and comprehensive treatment and long-term follow-up plan. Carrying out a comprehensive geriatric assessment in patients hospitalized for an acute disease is associated with a greater probability of survival and return home at discharge. It is associated with a reduction in costs compared to the usual control [20]. However, its value is unclear in patients with a recent HF hospitalization.

Several studies have shown that patients with HF treated by cardiologists have longer survival than those followed by non-cardiologists [21,22]. However, previous studies have also demonstrated cardiologists’ overuse of invasive treatments in frail patients in whom they will offer little benefit [23]. Considering the difficulty in assessing the presence of frailty, it would be expected that the collaboration between geriatricians and cardiologists improve the prognosis and quality of life of elderly patients with HF. This collaboration would allow identifying frail patients in whom invasive or aggressive treatment should be forgone, and at the same time, identify non-frail older patients that would benefit from all treatments and interventions available. Therefore, this study aimed to assess whether the combined follow-up by a geriatrician and a cardiologist after hospital admission for HF in older patients reduces 1-year all-cause hospitalization compared to follow-up by a cardiologist alone.

## 2. Materials and Methods

### 2.1. Study Design

This was a randomized controlled trial in which we consecutively included all patients aged 75 years or older admitted due to decompensated HF at the cardiology department of Hospital del Mar. All patients came from home, and none were admitted from a long-term care facility. The study was registered at ClinicalTrials.gov (number NCT03555318).

The main exclusion criteria were patients who declined to participate in the study, patients in palliative care or with a life expectancy of less than one year, patients who were referred to skilled nursing facilities or nursing homes at hospital discharge, patients with planned intervention on the mitral or aortic valve during admission or scheduled early after discharge, patients included in other randomized controlled trials, and patients who, at the investigator’s discretion, were considered unable to participate in the study. A patient was considered to have a life expectancy < 1 year if they were discharged to a hospice or to receive palliative care at home, or if other comorbidities (i.e., cancer) were in an advanced stage with a low likelihood of survival more than one year according to the respective specialist. In patients with planned intervention on the mitral or aortic valve, given that an intervention could successfully solve the main cause of heart failure, a geriatrician (and a cardiologist) might be less relevant. The main reasons to exclude patients at the investigator’s discretion were those who did not comply with medication intake or attended the Heart Failure Unit or primary care visits.

### 2.2. Study Procedures

The study procedures are summarized in Supplemental Table S1. Patients were randomized 1:1 to usual care follow-up (cardiologist from the Heart Failure Unit) (control group) or follow-up by a cardiologist and a geriatrician (group intervention). In the intervention group, the cardiologist and the geriatrician did the consultation simultaneously, both of them in the same room with the patient. Treatment changes were discussed between both specialists at that time. A geriatrician did not see patients in the cardiologist alone group. The consultation on the first and last visit lasted 1 h. The visits at 3 and 6 months lasted 30 min. All patients also had follow-ups by their primary care doctor and nurse within a heart failure care pathway. After an HF hospitalization, the patients’ primary physician was the cardiologist of the Heart Failure Unit. Therefore, primary care doctor visits were unrelated to heart failure. The Heart Failure Unit includes patients with HF irrespective of left ventricular ejection fraction.

Randomization was stratified according to the presence or absence of frailty at hospital discharge and ventricular function to ensure balanced groups. This stratification was achieved by generating four different randomization lists: one for frail patients (with and without preserved ventricular function) and another for patients without frailty (with and without preserved ventricular function). Frailty was assessed using the Canadian Study of Health and Aging (CSHA) Clinical Frailty Scale [24], and a patient was considered frail if their score was equal to or greater than 4. Preserved ventricular function was defined as an ejection fraction equal to or greater than 50%. Randomization was performed by independent study administrative staff following a computer-generated randomization scheme.

All visits were carried out at the Day Hospital of the Cardiology Department. The first outpatient visit was scheduled to occur less than 10 days after discharge from the hospital, and the other visits at 3, 6, and 12 months after discharge. This is the standard follow-up schedule in patients with HF followed at the Heart Failure Unit. Clinical events that happened during the duration of the study were adjudicated blindly.

The Ethics Committee approved the study (number 2017/7653/I), and all patients gave their written informed consent before being included in the study.

### 2.3. Variables

We collected sociodemographic and clinical variables, cardiovascular risk factors, comorbidities (Charlson index), time of evolution of heart failure (year of diagnosis and number of decompensations during the previous year), and usual treatment. A fasting blood test was performed to analyze the values of creatinine, glomerular filtration, hemogram, ferritin, transferrin saturation, total proteins, albumin, thyroid-stimulating hormone (TSH), liver function test, vitamin B12, folic acid, and vitamin D. On discharge day, an analysis of ultrasensitive troponin T and N-terminal prohormone of brain natriuretic peptide (NT-proBNP) values was performed. The ejection fraction was assessed by the echocardiogram performed during or within six months of the hospitalization. Anemia was defined by hemoglobin concentration < 12.0 g/dL in women and <13.0 g/dL in men. Chronic kidney disease (CKD) was defined by glomerular filtration rate (GFR) equal to or lower than 60 mL/min/1.73 m^2^ [25]. The presence of sleep apnea was defined as the use of continuous positive airway pressure (CPAP) or a diagnosis in the medical record based on a sleep study. A patient had peripheral vascular disease if documented in medical charts after evaluation by a vascular surgeon or by the presence of invasive or non-invasive tests documenting abnormal results.

Regardless of the group assigned, all patients were assessed with the Canadian Study of Health and Aging (CSHA) Clinical Frailty Scale during the hospitalization [24]. This scale was based on the patient’s status before the hospitalization. During the first visit and at one year of follow-up, we assessed functional status with the Lawton [26] and Barthel index [27], cognitive status with the Pfeiffer test [28], and quality of life with the European Quality of Life -5 Dimensions test (EQ-5D) and the Visual Analog Scale (VAS) [29] (both generic questionnaires on quality of life), and the Kansas City Cardiomyopathy Questionnaire (KCCQ) (specific for heart failure) [30]. In patients randomized to the intervention group, the following areas were assessed by the geriatrician: social sphere with the Gijón socio-family assessment scale (abbreviated and modified) (Barcelona version) [31], the emotional sphere with the Geriatric Depression Scale Yesavage [32], nutritional status with Mini-Nutritional Assessment Short Form [33] and plasma albumin, and the presence of geriatric syndromes (constipation, falls, pressure ulcers, polypharmacy, incontinence, insomnia).

### 2.4. Geriatrician’s Interventions

After the geriatrician assessment, interdisciplinary interventions were carried out in each area evaluated (Supplemental Table S2). Eighteen different interventions could be implemented depending on the patient’s needs: Functional rehabilitation program (home physiotherapy), physical exercise guidelines, education on improving the basic activities of daily living, fall prevention education, education on cognitive impairment, referral to the dementia outpatient clinic, health education on the management of delirium, the non-pharmacological and pharmacological treatment for insomnia, non-pharmacological and pharmacological treatment of depression, intervention on polypharmacy (review of medicines and deprescription), education of urinary incontinence, education of fecal incontinence, and intervention of constipation; education and intervention of nutritional status, administration of supplemental enteral nutrition, intervention in pressure ulcers, and intervention by a health social worker. Patients underwent a comprehensive geriatric assessment at baseline and 1-year follow-up. At months 3 and 6, the geriatrician assessed the improvement of the problems detected at baseline. A shorter, focused assessment of needs was performed during these consultations. Therefore, the geriatrician was able to act on any problem detected in each area evaluated during the whole duration of the study.

### 2.5. Study Outcome

The main aim was to evaluate whether the intervention of a cardiologist and a geriatrician reduces all-cause hospital admissions at one-year follow-up. Hospital admission was defined as any hospital admission or an emergency room stay of more than 18 h, including planned hospitalizations or interventions, even if they did not require an overnight stay. Hospitalizations were ascertained at each visit. Moreover, the electronic health system allows us to view any hospitalization carried out at any hospital within the public health system. Therefore, whenever a patient explained that they had been hospitalized, we had access to the discharge summary, tests, and procedures performed during the hospitalization. The use of the private health system in patients with heart failure is anecdotal in our setting [1]. Given the study design, it was impossible to assess the endpoints blinded. Although hospitalization is a hard endpoint, some of the secondary endpoints could be biased by the unblinded nature of the study.

The secondary objectives were to evaluate whether this intervention reduced hospital HF readmissions, reduced all-cause mortality, improved the quality of life, reduced polypharmacy, and maintained or improved functional capacity. As a non-prespecified analysis, we also analyzed planned and un-planned hospitalizations or procedures and emergency room visits. A combined outcome of all-cause hospitalization and all-cause death was also assessed. Two consultant cardiologists specialized in heart failure (NF, SRB) blindly adjudicated events.

### 2.6. Statistical Analysis

Sample size calculation was difficult since no studies have evaluated the effect of geriatric intervention in conjunction with cardiology in the follow-up patients with HF. However, multidisciplinary interventions in our setting have been shown to decrease clinically related readmission in patients with HF over 65 years of age by 31% [34]. Thus, we calculated that 114 patients in the conventional follow-up group and 114 patients in the joint follow-up group would be necessary to detect as statistically significant the difference between the two proportions (hospitalization for any cause at one year, estimated at 48% in the control group 1 and 30% in the intervention group [34]) accepting an alpha risk of 5% and a beta risk of less than 20% in a bilateral contrast using the ARCSINUS approach [35]. Therefore, taking into account the number of admissions for HF in our Cardiology Department, it was considered that it would be feasible to complete the inclusion of patients in 1 year. However, patients with exclusion criteria or who refused to participate were higher than expected, and therefore the estimated patient goal has not been reached. On the other hand, the number of events was much higher than expected.

Continuous variables were summarized as mean and standard deviation, and categorical variables as proportions. Chi-square test or Fisher’s exact test for categorical variables and Student *t*-test for continuous variables were used to assess the baseline differences. The primary analysis was performed according to the intention-to-treat principle. We included data from all patients who had undergone valid randomization in the primary and secondary outcomes analyses. Time-to-event data were evaluated using Kaplan–Meier estimates and Cox proportional-hazards models. We did not attempt a competing risk analysis because only one patient died without having a previous rehospitalization. Using the Cox models, hazard ratios, 95% confidence intervals, and two-sided *p* values were calculated.

Study data were collected and managed using REDCap electronic data capture tools hosted at Parc de Salut Mar [36,37]. REDCap (Research Electronic Data Capture) is a secure, web-based software platform designed to support data capture for research studies, providing (1) an intuitive interface for validated data capture; (2) audit trails for tracking data manipulation and export procedures; (3) automated export procedures for seamless data downloads to common statistical packages; and (4) procedures for data integration and interoperability with external sources.

## 3. Results

**Figure 1:**
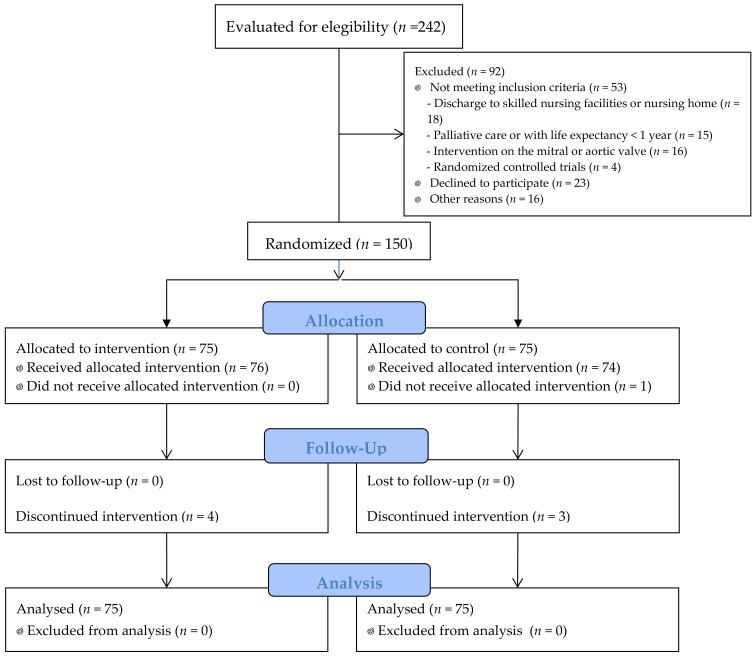
Flow diagram of the study.

One hundred and fifty patients were randomized between 2 July 2018 and 15 November 2019. The flow diagram of the study is summarized in Figure 1. Of the 242 patients assessed for eligibility, 38% could not be included in the study. The main reasons were patients who declined to participate (25% of patients not included) and discharge to skilled nursing facilities or nursing homes (19.6%).

Baseline characteristics are summarized in Table 1 and Table 2. Briefly, the mean age was 82.2 years (range 75–94 years), 50% were female, and 65.3% had heart failure with preserved ejection fraction (HFpEF). Comorbidities were highly prevalent, and 52% of patients were frail. Most of the patients were taking diuretics, and the use of proton pump inhibitors, benzodiazepines, and antidepressants was high. Groups were well balanced across baseline characteristics without differences between groups. In patients with HFrEF, 12 (36.4%) patients were taking sacubitril-valsartan, 7 (21.2%) angiotensin-converting enzyme inhibitors, and 3 (9.1%) angiotensin II receptor blockers; 13 (39.4%) were taking a mineralocorticoid receptor antagonists; 29 (87.9%) were under betablocker treatment and 3 (9.7%) received ivabradine. Only one patient (11.1%) was taking a sodium-glucose transporter type 2 inhibitor. However, it is worth noting that this drug group was not approved with an HF indication when the study was carried out. There were no statistical differences in HFrEF treatment between the usual care and intervention groups.

The first appointment was made a median (interquartile range) of 6 (5–9) days after discharge, without differences between groups. SARS-CoV-2 pandemic interfered with the study, and some 6-months and one-year follow-up visits had to be postponed. Therefore, one-year visits were made at a median (interquartile range) follow-up was 54 (49–61) weeks, without differences between groups. In the intervention group, patients with frailty received more frequent home physiotherapy, fall prevention education, education of urinary incontinence, and education and intervention of nutritional status.

**Figure 2:**
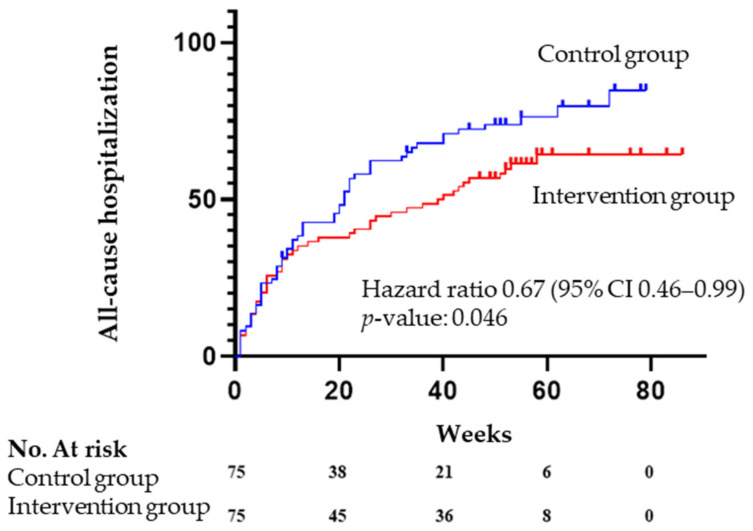
Kaplan–Meier curves for the primary endpoint, according to the study group.

Seventy percent of patients had at least one all-cause hospitalization during the study period. The primary outcome event occurred in 47 of 75 patients (62.7%) in the intervention group and in 58 of 75 patients (77.3%) in the control group (hazard ratio for all-cause hospitalization, 0.67; 95% confidence interval, 0.46 to 0.99; *p* = 0.046) (Table 3 and Figure 2).

Moreover, the time to the first hospitalization was longer in the intervention group (33.6 ± 25.3 weeks vs. 25.5 ± 22.6, *p* = 0.042). The number of patients with at least one HF hospitalization was similar in both groups (26 patients (34.7%) in the intervention group vs. 30 (40%) in the control group, *p* = 0.5). There were 236 hospitalizations during the study period. The main reasons for hospitalization were heart failure (90 hospitalizations, 38.1%) and infection (35 episodes, 14.8%). Only one patient was hospitalized due to SARS COVID-19 pneumonia. Planned interventions were the third reason for hospitalization (33 procedures, 13.9%). The most frequent reasons were non-cardiac surgery (16 procedures, 48.4%) and cardiac procedures: six patients received a pacemaker, cardiac resynchronization therapy with or without a cardioverter-defibrillator therapy, two patients had a percutaneous coronary intervention, one patient valve surgery, and one underwent transcatheter aortic valve implantation. The fourth reason for hospitalization was bleeding or anemization (22 episodes, 9.3%). During follow-up, patients spent a median of 17.5 days hospitalized (18 days (interquartile range: 4–28.5) in the intervention group and 16 (6–29) in the control group, *p* = 0.77.

Table 3 shows the emergency room visits, which were not different between groups. Twenty-one patients (14%) received at least one day of intravenous diuretics at the heart failure day hospital or local urgent primary care facilities (12% in the intervention group vs. 16% in the control group, *p* = 0.48).

Table 4 shows the prespecified group analysis. We found differences in sex, history of heart failure, and ejection fraction.

Thirty-seven patients died during the study period (24.7%). The leading cause of mortality was heart failure (20 patients, 54.1% of all deaths), followed by infection (13.5% of patients), and cancer (10.8%). Although there were no differences statistically differences in the cause of death between groups, heart failure was the cause of death in 64.7% of patients in the intervention group vs. 45% in the control group, infection 11.8% vs. 15%, cancer 11.8% vs. 10%, and other causes 11.8% vs. 30%, *p* = 0.54.

## 4. Discussion

In our study involving older patients with a recent heart failure hospitalization, a follow-up by a cardiologist and geriatricians was more effective in reducing the risk of all-cause hospitalization than was a follow-up by a cardiologist alone. However, the number of all-cause hospitalizations was extremely high, and one in four patients died at one year of follow-up.

The population of this study consisted of 50% female patients, with a mean age of 82 years old, and mostly HF with preserved ejection fraction. The most relevant finding is that baseline characteristics and comorbidities (mainly hypertension, anemia, and chronic kidney disease) are similar to other cohorts of older patients with HF [38,39,40,41]. Frailty was present in half of the patients, and only 28% were independent in basic activities of daily living, also consistent with previous studies [14,15].

We found an extremely high number of rehospitalization. Indeed, 70% of patients had an all-cause hospitalization during the one-year follow-up. This number is higher than previously published, where around 21.6 to 58.7% of patients were rehospitalized. However, it is worth mentioning that most of these studies included patients up to 10 years younger than ours [1,3,4,5]. HF rehospitalization is usually the leading cause of rehospitalization in HF patients, accounting for 13.1 to 20.1% of cases [3,42]. In our study, HF hospitalization was consistent with previous studies and was the leading cause of rehospitalization (38.1% of all hospitalizations). Another reason for the higher all-cause hospitalization rate we found, apart from age, comorbidities, and frailty, is that we included planned and unplanned hospitalizations and interventions. Interestingly, planned procedures or interventions were the third cause of hospitalization (13.9%). This reason for hospitalization is not usually contemplated in studies on HF patients. However, considering how frequent it is and the effect a geriatrician assessment can have on setting the indication of a procedure considering risk/benefit according to baseline frailty, planned procedures or interventions should be included in studies focusing on older comorbid patients. Briefly, focusing on a particular reason for hospitalization might be irrelevant in older HF patients since they are often multifactorial. Moreover, identifying patients with frailty in which invasive interventions (cardiac or non-cardiac) would be futile would improve overall care and potentially decrease healthcare costs. Finally, hospitalization due to COVID-19 was highly infrequent. Several studies have shown that the prevalence of heart failure in hospitalized patients with COVID-19 is low [43]. One possible explanation is that patients would have been especially careful in self-isolating since it is an older population with higher baseline risk. Thus, their risk of infection might have been lower than in the general population.

A comprehensive geriatric assessment carried out by a geriatrician can identify frail patients and other domains and geriatric syndromes that can be addressed. In this study, the intervention of a geriatrician was associated with a 33% decrease in all-cause hospitalization at one year (hazard ratio 0.67, 95% confidence interval 0.46–0.99, *p* = 0.046). Moreover, the time to the first hospitalization was almost eight weeks longer in the intervention group (33.6 ± 25.3 weeks vs. 25.5 ± 22.6, *p* = 0.042). The highest risk for a new heart failure hospitalization after a previous one is during the first three months of discharge. Therefore, in this vulnerable period, the cardiologist’s role might be more important by trying to avoid a new decompensation. After this high-risk period, other comorbidities can complicate the medical course, and the geriatrician role might be more relevant. A randomized study of a nurse-coordinated cardiac care bridge transitional care program after a cardiovascular hospitalization did not reduce hospital readmission or mortality within 6 months. However, most of the interventions were focused on coordinating care between hospital and primary care, and HF patients only comprised 58% of the patients included [44].

One of the strongest points of the study is that the consultation was made at the same time by the cardiologist and geriatrician. This allowed discussion of any potential changes in treatment or interventions at the time of the visit and decrease the number of visits per patient because it was not necessary to go to the cardiologist and geriatrician on different days or hours. The consultations were time-consuming and lasted longer than is usual. However, the improvement in outcomes strongly suggest that the way we routinely provide care to frail patients should be modified. The small sample limits subgroup analysis, but it would appear that women, patients with HF with preserved ejection fraction, and those with a previous history of HF would benefit the most.

Apart from the study visits that were carried out at the Day Hospital, 48% of patients were followed up using several types of telemedicine (i.e., structured telephone follow-up or non-invasive devices), which are the standard follow-up options in our center [45]. There was no difference in the type of follow-up between groups. None of the patients was followed up with invasive monitoring (CardioMEMS^®^). In the future, artificial intelligence-based systems might help us identify the patients more likely to benefit from this intervention [46].

Finally, we found no difference in mortality between both groups. At one-year follow-up, 24.7% died. This rate was similar to other series that included younger patients [4,5] but lower than other studies on older (>75 years old) patients [39,41]. As expected, the main cause of death was HF. Interestingly, although the cause of death was not statistically different between groups, the intervention group had more heart failure as the cause of death and far fewer other causes than the control group. It is plausible to think that the geriatrician intervention helped decrease the non-HF cause of death, thus making HF the cause of death in 64.7% of patients who died in the intervention group.

### Limitations

The number of patients excluded was higher than expected. Discharge to skilled nursing facilities or nursing homes was one of the main reasons (7.4% of the total patients evaluated). In this group of patients, a worse prognosis has been described that is frequently not related to their cardiac pathology [47], so we consider that their inclusion could distort the results. SARS-CoV-2 epidemic impeded some follow-up appointments to be carried out in a timely matter according to protocol, and some patients had the last follow-up visit months after the one-year mark. Moreover, some geriatric interventions, such as day-care and rehabilitation, could not be carried out during this time. Finally, the results need to be confirmed in other settings with different follow-up arrangements.

## 5. Conclusions

In older patients with a recent HF hospitalization, a follow-up by a cardiologist and geriatrician was superior to a cardiologist’s follow-up in reducing all-cause hospitalization.

## Figures and Tables

**Table 1:** Baseline characteristics and medication at hospital discharge of the patients included in the study.

	Usual Care (*n* = 75)	Intervention (*n* = 75)	*p*-Value
Age (years)	82.6 ± 4.5	81.6 ± 4.9	0.22
Female	38 (50.7)	37 (49.3)	0.87
Hypertension	67 (89.3)	66 (88)	0.59
Diabetes mellitus	32 (42.7)	32 (42.7)	0.82
Dyslipidemia	49 (65.3)	46 (61.3)	0.61
Stroke/TIA	9 (12)	10 (13.3)	0.88
Chronic kidney disease	54 (72)	52 (69.3)	0.72
Anemia	48 (64)	42 (56)	0.32
Sleep apnea	8 (10.7)	8 (10.7)	0.98
Peripheral vascular disease	10 (13.3)	14 (18.7)	0.41
Chronic lung disease	28 (37.3)	20 (26.7)	0.16
Cancer	19 (25.3)	18 (24)	0.92
Myocardial infarction	21 (28)	15 (20)	0.25
Coronary intervention	16 (21.3)	13 (17.3)	0.54
TAVI or Mitraclip	1 (1.4)	3 (4)	0.51
Cardiac surgery:			
- CABG - Valve replacement - CABG and valve replacement	2 (2.7) 3 (4) 1 (1.4)	3 (4) 7 (9.3) 3 (4)	0.37
Atrial fibrillation or flutter	47 (62.7)	57 (76)	0.08
Moderate to severe valve disease	25 (33.3)	24 (32)	0.86
Device therapy:			
- Pacemaker - CRT or ICD	9 (12) 3 (4)	14 (18.7) 2 (2.7)	0.19
Previous history of HF	42 (56)	43 (57.3)	0.87
Duration of HF *:			
- <3 months - 3–6 months - 6–12 months - 1–5 years - >5 years	7 (16.3) 3 (7) 6 (14) 22 (51.2) 5 (11.6)	9 (20.5) 1 (2.3) 3 (6.8) 14 (31.8) 16 (36.4)	0.06
HF hospitalization the previous year *	19 (45.2)	15 (36.6)	0.42
HF categories:			
- HFpEF (LVEF ≥ 50%) - HFmrEF (LVEF 40–49%) - HFrEF (LVEF < 40%)	47 (62.7) 11 (14.7) 17 (22.7)	51 (68) 16 (21.3) 8 (10.7)	0.72
Echocardiographic parameters			
LVEF (%)	51.3 ± 14.4	53.5 ± 13.9	0.34
TAPSE (mm), *n* = 138	17.9 ± 4.0	17.2 ± 3.9	0.30
Left ventricular mass index (g/m^2^). *n* = 145	129.9 ± 35.8	127.3 ± 33.7	0.96
Right ventricle (mm), *n* = 95	28.1 ± 7.0	30.4 ± 6.6	0.09
Medications at discharge			
ACEI/ARB-2/ARNI	35 (46.7)	43 (57.3)	0.19
MRA	9 (12)	12 (16)	0.48
Betablockers	55 (73.3)	53 (70.7)	0.72
Diuretics	71 (94.7)	73 (97.3)	0.34
Anticoagulation	48 (64)	58 (77.3)	0.07
Antiplatelet therapy	16 (21.3)	15 (20)	0.84
Oral antidiabetic drugs	24 (32)	24 (32)	0.96
Insulin	14 (18.4)	14 (18.4)	1.0
Proton-pump inhibitors	58 (77.3)	47 (62.7)	0.05
Statin	52 (69.3)	44 (58.7)	0.17
Calcium channel antagonists	23 (31.3)	21 (28.4)	0.72
Nitrates	17 (22.7)	14 (18.7)	0.55
Hydralazine	9 (12)	12 (16)	0.48
Amiodarone	12 (16)	13 (17.6)	0.80
Digoxin	2 (2.7)	2 (2.7)	0.69
Vitamin D supplements	25 (33.3)	21 (28)	0.48
Oral iron supplements	21 (28)	21 (28)	1.0
Benzodiazepines	17 (22.7)	13 (17.3)	0.41
Antidepressant drugs	16 (21.3)	23 (30.7)	0.19
Bronchodilators	28 (37.3)	22 (29.3)	0.30

Data are number (percentage) or mean ± standard deviation. ACEI: angiotensin-converting enzyme inhibitors. ARB-II: angiotensin II receptor blockers. ARNI: angiotensin receptor and neprilysin inhibition. CABG: Coronary artery bypass grafting. CRT: Cardiac Resynchronization Therapy. CKD: Chronic kidney disease. HF: Heart failure. HFrEF: Heart failure with reduced ejection fraction. HFmrEF: Heart failure with mildly reduced ejection fraction. HFpEF: Heart failure with preserved ejection fraction. ICD: Implantable cardioverter-defibrillator. LVEF: Left ventricular ejection fraction. MRA: mineralocorticoid receptor antagonists. TAPSE: Tricuspid Annular Plane Systolic Excursion. TIA: transient ischemic attack. TAVI: Transcatheter Aortic Valve Implantation. * only for patients with a previous history of HF.

**Table 2:** Hospitalization and first appointment characteristics.

	Usual Care (*n* = 75)	Intervention (*n* = 75)	*p*-Value
Length of hospitalization (days)	9 (6–12)	9 (5–13)	0.71
NT-proBNP at discharge, pg/mL	2843 (1162–5943)	2454 (1456–4328)	0.51
High-sensitivity T troponin (Hs-TnT) at discharge, ng/L	46.29 (29.55–74.99)	37.18 (27.81–63.5)	0.15
eGFR (mL/min) at discharge	44.2 ± 19.5	48.5 ± 20.4	0.19
Days from discharge to the first appointment	6 (5–11)	7 (6–12)	0.08
Type of follow-up:			
- In-person at Day Hospital - Structured phone follow-up - Non-invasive telemedicine (devices)	36 (48) 20 (26.7) 14 (18.7)	42 (56) 10 (13.3) 14 (18.7)	0.16
Frailty (Clinical Frailty Scale) ≥ 4	39 (52)	39 (52)	1
Clinical Frailty Scale	4.1 ± 1.4	3.9 ± 1.2	0.49
Barthel index	84.0 ± 19.8	87.9 ± 13.4	0.17
Basic activities of daily living (Barthel index):			
- Independent (100) - Minimally dependent (61–99) - Partially to totally dependent (0–60)	22 (31.4) 38 (54.3) 10 (14.3)	20 (27.8) 49 (68.1) 3 (4.2)	0.073
Instrumental activities of daily living (Lawton index)	5.2 ± 2.3	4.7 ± 1.9	0.19
Pfeiffer Short Portable Mental Status Questionnaire (SPMSQ)	1.33 ± 1.4	1.76 ± 1.8	0.11
NYHA functional class	2.3 ± 0.6	2.2 ± 0.6	0.35

Data are number (percentage), mean ± standard deviation, or median (interquartile range). eGFR: estimated glomerular filtration rate. NT-proBNP: N-terminal prohormone of brain natriuretic peptide. NYHA: New York Heart Association.

**Table 3:** Primary and secondary outcomes during follow-up.

	Usual Care (*n* = 75)	Intervention (*n* = 75)	Hazard Ratio	*p*-Value
All-cause hospitalization	58 (77.3)	47 (62.7)	0.67 (0.46–0.99)	0.046
HF hospitalization	30 (40)	26 (34.7)	0.79 (0.46–1.33)	0.37
Planned intervention/ hospitalization	11 (14.7)	17 (22.7)	1.48 (0.69–3.20)	0.315
All cause hospitalization or death	60 (80.0)	47 (62.7)	0.67 (0.45–0.98)	0.038
All-cause mortality	20 (26.7)	17 (22.7)	0.81 (0.43–1.56)	0.53
Emergency room visit	35 (46.7)	32 (42.72)	0.80 (0.492–1.296)	0.36

Data are numbers (percentage). HF: Heart failure.

**Table 4:** Prespecified subgroup analyses.

	Intervention	Control	HR	CI 95	*p*
Overall	47/75	58/75	0.67	0.46–0.99	0.046
Sex					
Female	20/37	33/38	0.44	0.25–0.78	0.005
Male	27/38	25/37	1.02	0.59–1.76	0.94
Left ventricular ejection fraction					
Preserved	25/47	40/46	0.41	0.24–0.67	<0.001
Reduced	19/23	18/29	1.59	0.83–3.04	0.16
Frailty					
Yes	24/39	31/39	0.70	0.41–1.19	0.19
No	23/35	26/35	0.70	0.40–1.24	0.22
Previous HF diagnosis					
Yes	17/32	25/33	0.49	0.26–0.92	0.026
No	30/43	33/42	0.81	0.49.1.33	0.41
HF hospitalization in ≤12 months					
Yes	16/19	11/15	1.12	0.51–2.45	0.78
No	17/27	17/23	0.70	0.35–1.37	0.30

## Data Availability

The data presented in this study are available on request from the corresponding author. The data are not publicly available due to ethical restrictions.

## Supplementary Materials

The following supporting information can be downloaded at: https://www.mdpi.com/article/10.3390/jcm11071932/s1, Table S1: Schedule of events. Table S2: Geriatrician assessment and interdisciplinary interventions carried out in each area evaluated at the baseline visit.
