# Supplementary material for: Randomized Controlled Trial Comparing a Multidisciplinary Intervention by a Geriatrician and a Cardiologist to Usual Care after a Heart Failure Hospitalization in Older Patients: The SENECOR Study
Source: J Clin Med. 2022 Mar 30;11(7):1932. doi: 10.3390/jcm11071932 (PMC8999953; doi:10.3390/jcm11071932)
Supplement: Supplementary file 1 [file jcm-11-01932-s001.zip › jcm-1626424-supplementary.pdf]

Journal of
Clinical Medicine

MDPI

Supplementary Material: Randomized Controlled Trial Comparing a Multidisciplinary Intervention by a Geriatrician and a Cardiologist to Usual Care after a Heart Failure Hospitalization in Older Patients: The SENECOR Study

Marta Herrero-Torrus, Neus Badosa, Cristina Roqueta, Sonia Ruiz-Bustillo, Eduard Solé-González, Laia C. Belarte-Tornero, Sandra Valdivielso-Moré, Olga Vázquez and Núria Farré

Table S1. Schedule of events.

	Pre-Discharge	Baseline Visit	Month 3	Month 6	Month 12
Written informed consent	x				
Canadian Study of Health and Aging Clinical Frailty Scale	x				
Echocardiogram	x				
Electrocardiogram	x				
Blood test [1]	x				x
Lawton test	x	x			x
Barthel test	x	x			x
Pfeiffer test	x	x			x
Medical history and baseline characteristics		x			
Geriatrician assessment (intervention group only) [2]		x			x
Quality of life (EQ-5D, VAS, KCCQ)		x			x
Current medication		x	x	x	x
Physical examination, weight, blood pressure, heart rate, SpO2		x	x	x	x
NYHA functional class		x	x	x	x
Hospitalization, ED visit or IV ambulatory diuretics		x	x	x	x

[1] Blood test: creatinine, glomerular filtration, hemogram, ferritin, transferrin saturation, total proteins, albumin, thyroid-stimulating hormone (TSH), liver function test, vitamin B12, folic acid, and vitamin D. On discharge day, an analysis of ultrasensitive troponin T and N-terminal prohormone of brain natriuretic peptide (NT-proBNP). [2] Gijón socio-family assessment scale (abbreviated and modified) (Barcelona version), Geriatric Depression Scale Yesavage, Mini-Nutritional Assessment Short Form, presence of geriatric syndromes (constipation, falls, pressure ulcers, polypharmacy, incontinence, insomnia). ED: Emergency room, EQ-5D: European Quality of Life -5 Dimensions test, IV: intravenous, NYHA: New York Heart Association, KCCQ: Kansas City Cardiomyopathy Questionnaire (KCCQ), SpO2: Oxygen saturation, VAS: Visual Analog Scale.

Table S2. Geriatrician assessment and interdisciplinary interventions carried out in each area evaluated at the baseline visit.

Sphere or Geriatric Syndrome Assess	Type of Intervention	Number of Patients (%) Who Received the Intervention
Social sphere	Intervention by a health social worker	1 (1.4)
Emotional sphere	Pharmacological treatment of depression	7 (9.9)
Nutritional status	Education and intervention of nutritional status	43 (60.6)
	Administration of supplemental enteral nutrition	1 (1.4)
Daily living activities	Functional rehabilitation program (home physiotherapy)	15 (21.1)
	Physical exercise guidelines	21 (30)
	Education of improving the basic activities of daily living	3 (4.2)
Constipation	Intervention of constipation	14 (19.7)
Falls	Fall prevention education	20 (28.2)
Pressure ulcers	Intervention in pressure ulcers	0 (0)
Polypharmacy	Review of medicines and deprescription	11 (15.5)
Incontinence	Education of urinary incontinence	25 (35.2)
	Education of fecal incontinence	2 (2.8)
Insomnia	Non-pharmacological treatment for insomnia	19 (26.8)
	Pharmacological	5 (7)
Dementia/cognitive impairment	Education on cognitive impairment	4 (5.6)
	Referral to the dementia outpatient clinic	1 (1.4)
	Health education on the management of delirium	5 (7.1)

Data are based on the 71 patients that completed the baseline visit.
